# Left-Sided Gallbladder: Tips and Tricks to Safe Cholecystectomy

**DOI:** 10.7759/cureus.76503

**Published:** 2024-12-28

**Authors:** Nicole Hawkins, Matan Ben David

**Affiliations:** 1 General Surgery, Townsville University Hospital, Townsville, AUS; 2 Hepatobiliary Surgery, Mater Misericordiae University Hospital, Townsville, AUS; 3 Hepatobiliary Surgery, Townsville University Hospital, Townsville, AUS

**Keywords:** benign gallbladder diseases, biliary colic pain, difficult cholecystectomy, laporoscopic cholecystectomy, true left-sided gallbladder

## Abstract

Left-sided gallbladder (LSGB) is a rare anatomical variation where the gallbladder is to the left of the falciform ligament and ligamentum teres. Most commonly, it is discovered as an incidental finding at the time of operation (typically for cholecystectomy). We describe a case of left-sided gallbladder in a 71-year-old female. The patient presented with complaints of pain in the right upper quadrant and epigastric area, which had persisted for two months, accompanied by intermittent biliary colic over the previous five years. An ultrasound demonstrated a single 39 mm gallstone. She underwent an elective laparoscopic cholecystectomy, during which an incidental discovery of an LSGB occurred. Patients with LSGB have a relatively high risk of complications when proceeding with operative interventions, likely secondary to concurrent biliary and arterial anatomical variants. Images of the anatomy of LSGB and possible adaptations to the usual laparoscopic cholecystectomy techniques necessary to perform a safe operation are discussed, including division of the falciform ligament and altered port placements. LSGB is a rare anatomical variation that increases surgical risk at the time of cholecystectomy. Understanding this variation in anatomy is critical to undertaking safe operative interventions in these patients.

## Introduction

Left-sided gallbladder (LSGB) is a rare anatomical variant, with an estimated incidence of 0.1-1.2% [1]. Typically, the gallbladder is located beneath hepatic segments four and five of the liver, with the gallbladder fossa lying on the von Rex Cantlie line, which divides the liver into its left and right lobes. This is typically to the right of the ligamentum teres, which normally arises between the left lateral sector of the liver and segment four [2]. True LSGB is defined as a gallbladder below segment III of the liver and to the left of the ligamentum teres in the absence of situs inversus viscerum [3].

In LSGB, laparoscopic cholecystectomy is considered the standard treatment [3,4]. Patients with LSGB tend to present with typical biliary colic and cholecystitis symptoms [5], and the usual preoperative imaging (ultrasound or computerised tomography) commonly does not diagnose this anatomical variant. As such, most patients with LSGB are diagnosed intra-operatively, and so it often presents as an unexpected challenge to the surgeon [4,6]. LSGB is associated with higher rates of morbidity at cholecystectomy [4,7,8]. As such, it is imperative that general surgeons can identify this pathology at the time of laparoscopic cholecystectomy and have knowledge of operative techniques to complete cholecystectomy safely.

## Case presentation

The patient, a 71-year-old female, complained of intermittent right upper quadrant and epigastric pain that had persisted for two months, accompanied by infrequent episodes of biliary colic over the previous five years. She was otherwise well-integrated into the community, maintained a good functional status, and had no history of previous abdominal surgeries.

**Figure 1:**
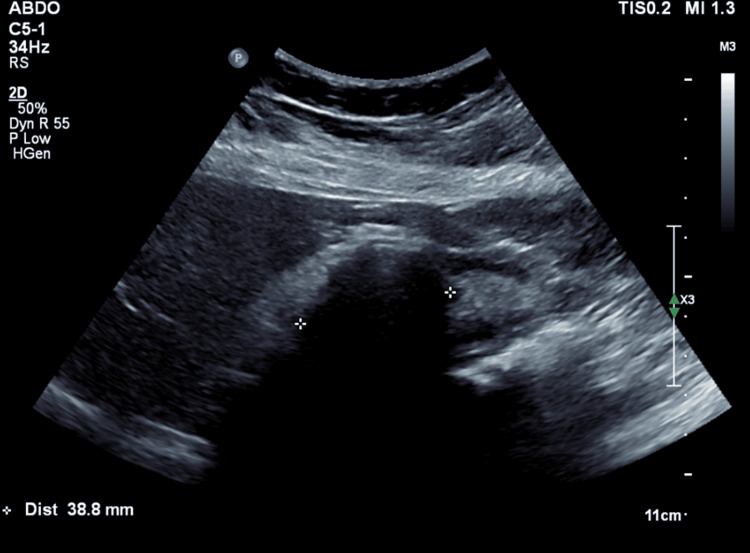
Ultrasound demonstrating a large 39 mm gallstone in the gallbladder, there is an acoustic shadowing typical of gallstones.

**Figure 2:**
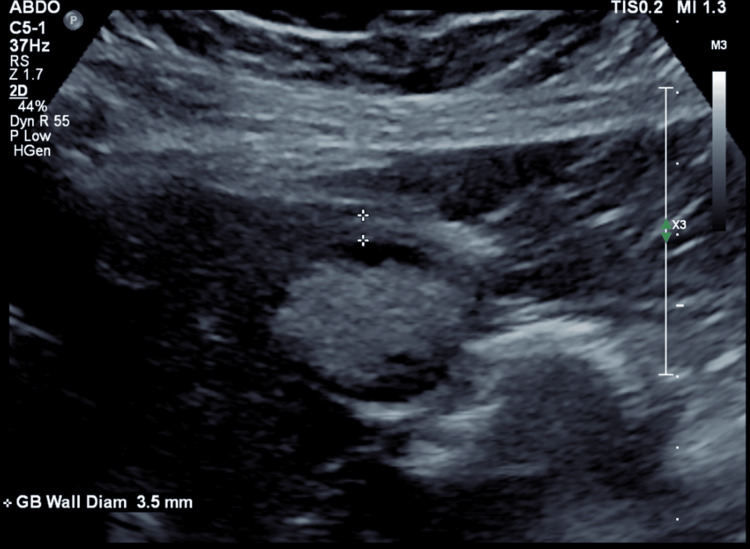
Ultrasound shows gallbladder wall thickness 3.5 mm, suggesting no signs of acute inflammation.

**Figure 3:**
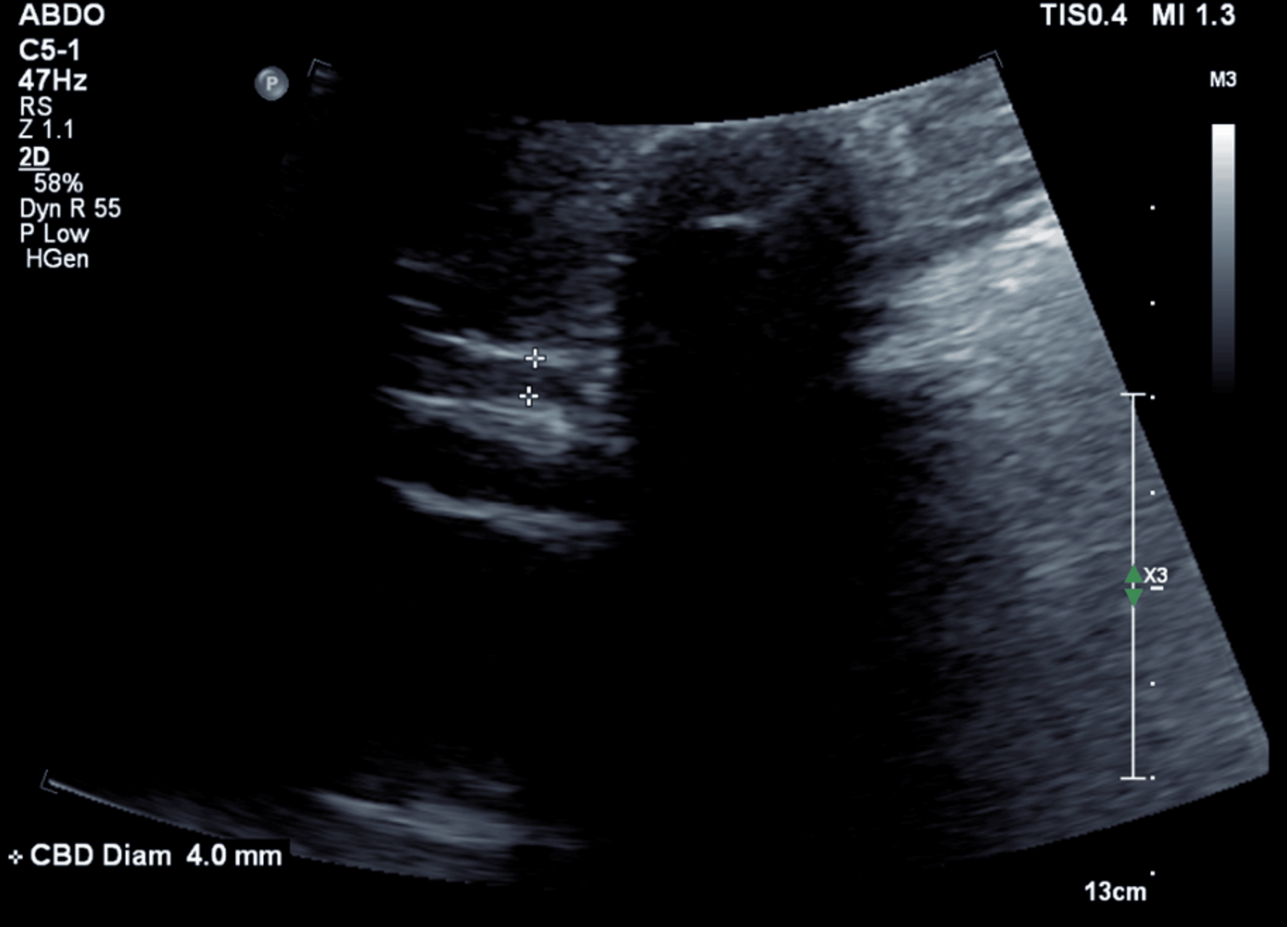
Ultrasound demonstrates a common bile duct diameter of 4.0 mm, consistent with a non-dilated biliary system.

An ultrasound demonstrated a single 39-mm gallstone with no signs of acute inflammation or dilated biliary system (Figures 1-3). Pre-operative blood was unremarkable (Table 1). She proceeded to an elective laparoscopic cholecystectomy for presumed biliary colic.

**Table 1:** Preoperative blood tests were consistent with biliary colic without any concerns for acute inflammation, infection, or obstruction. ALT: alanine transaminase; AST: aspartate aminotransferase; ALP: alkaline phosphatase; GGT: gamma-glutamyltransferase.

Test	Result	Units	Reference iInterval
Hb	156	g/L	110–160
WCC	8.3	10^9^/L	3.5–10.0
CRP	2.6	mg/L	<5
Bilirubin	6	μmol/L	<16
ALP	81	U/L	30–115
AST	27	U/L	10–35
ALT	39	U/L	5–30
GGT	34	U/L	5–35

**Figure 4:**
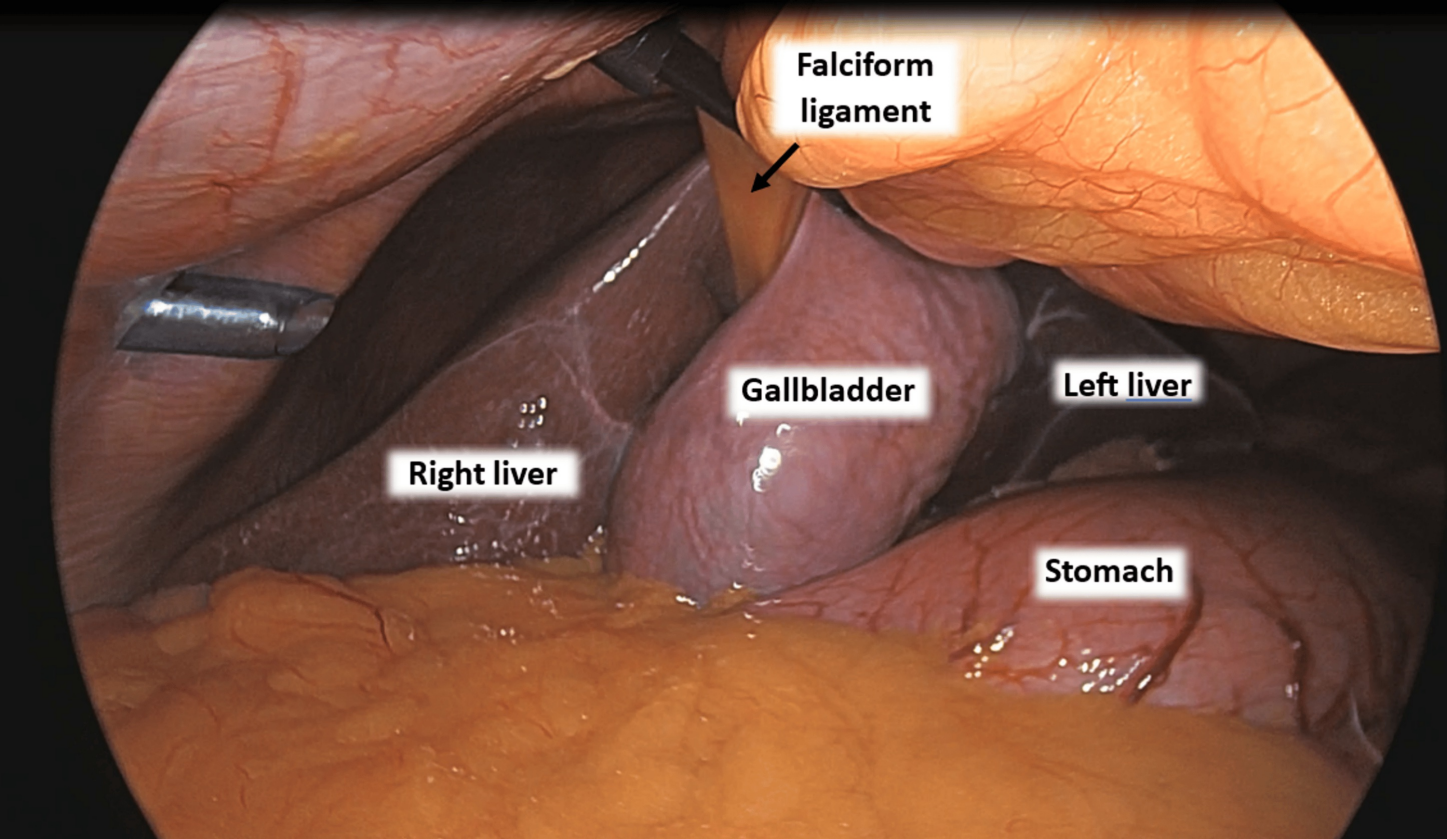
Anatomy of left-sided gallbladder. Note the gallbladder is to the left of the falciform ligament and under segment III of the liver.

After placement of the working ports in the “4 ports standard technique” - 10 mm port peri-umbilically, 5 mm port epigastric region, 5 mm port right lumbar region, and 5 mm port right upper quadrant - she was discovered to have an LSGB (Figure 4). Rather than re-site any of the ports, the decision was made to divide the falciform ligament to increase surgical exposure.

**Figure 5:**
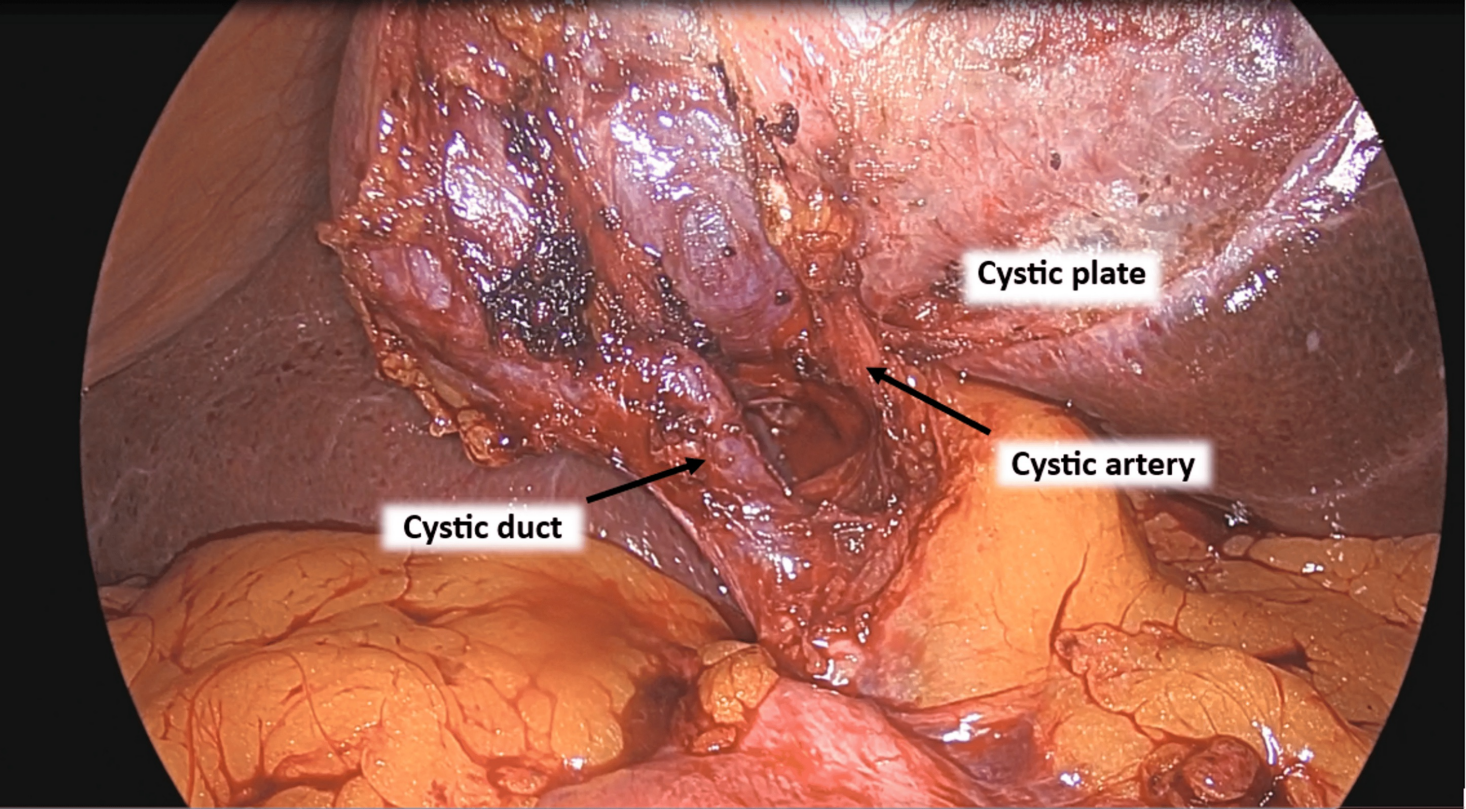
Critical view of safety established during laparoscopic cholecystectomy.

**Figure 6:**
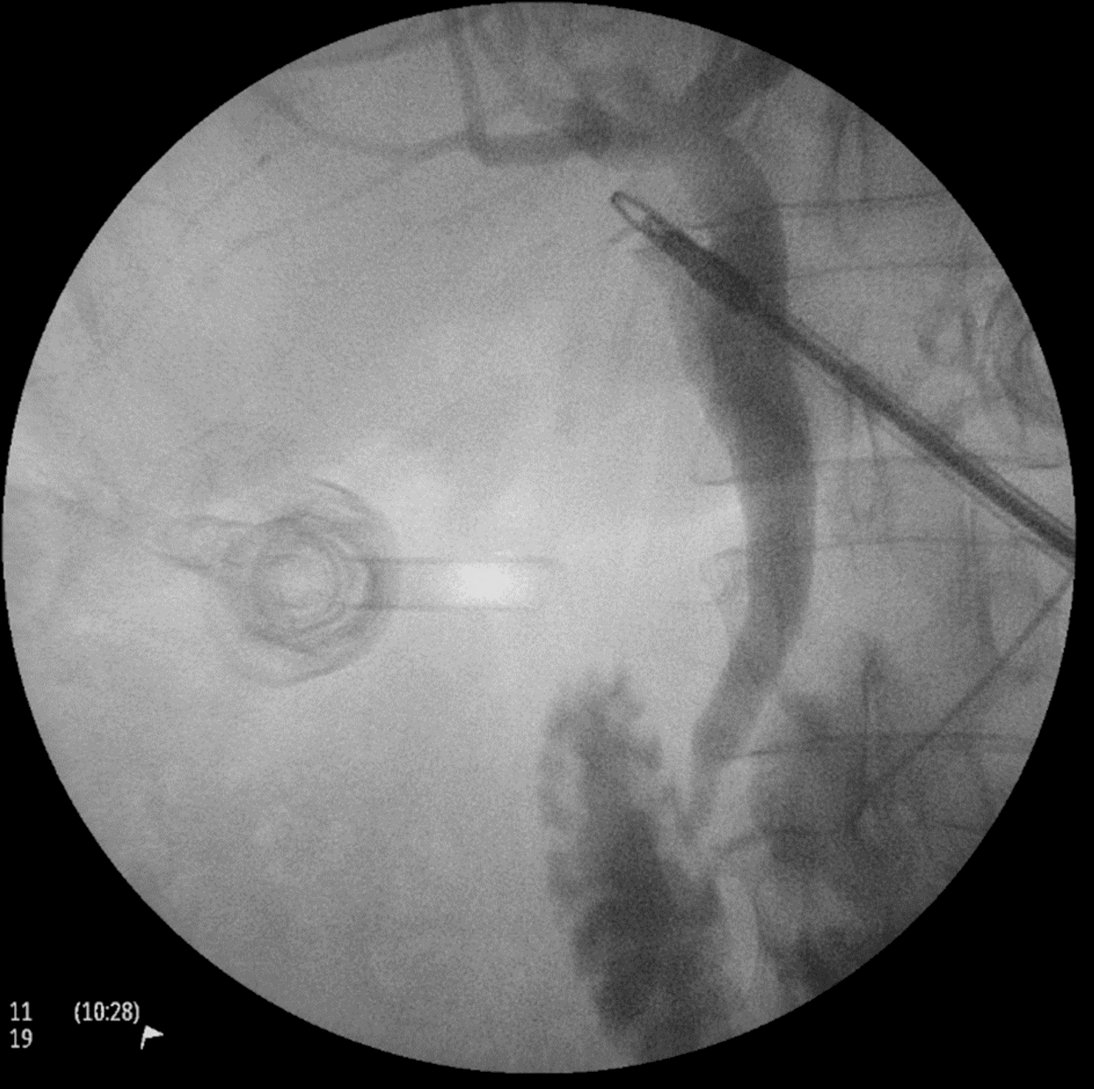
Intra-operative cholangiogram establishing normal biliary anatomy, good flow of contrast into duodenum with no evidence of obstruction.

By establishing the critical view of safety, we were able to perform the cholecystectomy (Figure 5). An intra-operative cholangiogram was performed, which did not demonstrate any aberrant anatomy (Figure 6). Histology confirmed chronic inflammation but showed no evidence of dysplasia or malignancy.

## Discussion

LSGB is one of many possible anatomical variations in the biliary tree [3]. Gros [9] proposes two embryological theories for its emergence. The first suggests the gallbladder develops as usual from a hepatic diverticulum; however, it migrates to the left side after attaching to the developing left lobe. This is associated with a long cystic duct that crosses the common hepatic duct, such that it joins the common hepatic duct from the right. The second theory suggests the gallbladder develops directly from the left hepatic duct with regression of the main gallbladder [9].

While no aberrant anatomy was identified in our case, bile duct and portal vein anomalies have commonly been reported in LSGB [4,6,7]. Variations in portal vein anatomy, although important in liver surgery and transplantation, are of limited clinical concern in laparoscopic cholecystectomy [7]. However, the rate of bile duct injury (BDI) is 4.4% in patients with LSGB who undergo laparoscopic cholecystectomy [4], which is higher than the estimated rate of 0.4-1.5% in the general population [10]. Theoretically, this high rate of BDI could potentially correlate with a higher rate of biliary variation. According to Gross's first theory, the most common variation occurs when the cystic duct crosses the hepatic duct and joins the common hepatic duct from the right.

Most cases of LSGB are found incidentally at the time of operation rather than preoperative imaging, as in this case. However, imaging can detect LSGB in some cases, with CT having a greater positive predictive value compared to ultrasound: 60% compared with 2.7%, respectively [4].

As such, LSGB is often an unexpected challenge encountered at the time of laparoscopic cholecystectomy. Various alterations to the “4 port standard technique” for laparoscopic cholecystectomy have been described to improve the exposure of LSGB and facilitate safe cholecystectomy. These include the resiting of the epigastric point to Palmer’s point or redirecting the epigastric port to enter to the left of the falciform ligament [11]; moving the right lumbar retraction port more medially and the epigastric/right-hand operating port to the left of midline [12]; placement of additional ports [13]; and mirror image setup, positioning ports to the left of midline [14].

As port placement was completed before discovering the patient had LSGB in this case, the decision was made to proceed with traditional port placement and division of the falciform ligament. This approach has previously been described by Malla et al., who also encountered no complications [15]. Alternatively, manipulation and retraction of the falciform without division have also been described to successfully complete cholecystectomy [16].

An intraoperative cholangiogram is generally recommended to define biliary tree anatomy, screen for anatomical anomalies, and ensure no BDI has occurred [14,17]. While some emphasise the need for meticulous dissection and establishment of the critical view of safety [18], as is standard for laparoscopic cholecystectomy [19], an antegrade or “fundus-first” approach to dissection has also been described for LSGB [20]. When concerns for aberrant anatomy arise or the critical view of safety cannot be established, however, consideration to convert to an open procedure is strongly advocated for by some [8,18], likely because of the relatively high rate of BDI in patients with LSGB.

## Conclusions

LSGB, a rare anatomical variation, frequently poses an unexpected challenge for general surgeons. We present a case of unexpected intraoperative LSGB, which we successfully managed laparoscopically. Cholecystectomy has a higher rate of morbidity in LSGB, and a review of the literature suggests many possible adjuncts to standard techniques to complete cholecystectomy safely. These include altered port placement, additional port placement, manipulation or division of the falciform ligament, and a low threshold to convert to open. By dividing the falciform ligament to increase surgical exposure, we were able to perform laparoscopic cholecystectomy with standard port placement, thereby facilitating the establishment of the critical view of safety. It is crucial for general surgeons to identify LSGB during cholecystectomy and utilize techniques to enhance surgical exposure.
